# Provider perspectives on adolescent access to contraception in South Africa

**DOI:** 10.4102/phcfm.v16i1.4537

**Published:** 2024-07-16

**Authors:** Thabile J. Ketye, Gbotemi B. Babatunde, Solange Mianda, Olagoke Akintola

**Affiliations:** 1 School of Public Health, Faculty of Community and Health Sciences, University of the Western Cape, Cape Town, South Africa; 2 Graduate School of Professional Psychology, University of Denver, Denver, United States

**Keywords:** access, adolescents, clinics, contraceptive methods, healthcare providers, sexual and reproductive health, South Africa

## Abstract

**Background:**

Poor access to contraception influences adolescent health outcomes and may lead to sexual and reproductive health challenges. Unmet sexual and reproductive health should contribute to unplanned adolescent pregnancies, sexually transmitted infections, and other conditions. Therefore, it is crucial to enable adolescents to access appropriate contraceptive methods easily.

**Aim:**

This study explored factors influencing adolescents’ access to contraceptive methods from the perspective of primary healthcare providers in South Africa.

**Setting:**

This study was conducted in two health districts of the Western Cape province in South Africa: the City of Cape Town Metropolitan Municipality and the West Coast district.

**Methods:**

Using a qualitative research design, in-depth semi-structured interviews were conducted with 24 healthcare providers who work in primary healthcare clinics. Subsequently, the interviews were transcribed and verified for errors. Braun and Clarke’s thematic analysis model guided the data analysis using ATLAS.ti software (version 22). The study adhered to the consolidated criteria for reporting qualitative studies checklist for qualitative research.

**Results:**

Four themes emerged through the data analysis: (1) personal influences, (2) community-level influences, (3) health system influences, and (4) policy-level influences. Representative quotations were used to illustrate the themes and sub-themes.

**Conclusion:**

Adolescence is shaped by various influences that affect adolescents’ ability to access contraception. These factors include their awareness of contraception, social environment, provider biases and school policies. Understanding these influences is crucial for addressing unintended pregnancies and promoting sexual and reproductive health among this age group.

**Contribution:**

This study highlights strategies that facilitate and hinder adolescents’ access to contraception.

## Introduction

Adolescents constitute 1.6 billion of the world’s population.^1^ This period, which falls between the ages of 10 and 19, is one of physical transition and development and of exploration and learning to be responsible.^2^ During this period, young people develop intimate bonds and learn to enjoy the pleasures of sexual activity, which may lead to unintended pregnancies. Some sexual and reproductive health (SRH) problems experienced during adulthood begin during adolescence.^3^ Among these are sexually transmitted infections (STIs), including the human immunodeficiency virus (HIV).

The incidence of adolescent pregnancies has increased in some parts of South Africa.^4,5^ This may have been exacerbated by the coronavirus disease 2019 (COVID-19) pandemic.^5,6^ Childbearing rises rapidly with age, from 4% among girls aged 15 to 28% among girls aged 19.^7^ This is despite the provision of effective contraceptive methods at no cost to anyone from the age of 12 years at South African public clinics.^8^ Effective contraceptive methods include condoms, oral contraceptive pills, injectables, and implants.^8^ These are accessed through healthcare providers who are trained in family planning (or contraception), such as nurses.^9,10^ Thus, providers play an integral role in facilitating adolescent access to contraception.

Adolescents’ access to contraceptive methods is crucial to prevent unintended pregnancies, STIs, and other preventable conditions.^11^ Levesque et al. state that access is possible when the identified needs of healthcare users are met through affordable, reachable, appropriate, and utilised services.^12^ Thus, adolescents’ access to contraceptive methods can be defined as the adolescents’ capacity to obtain appropriate methods in response to their healthcare needs.^12^ Access to healthcare is a function of enabling and impeding factors and can be illustrated through the utilisation of healthcare services.^13^

According to the United Nations (UN) Sustainable Development Goal (SDG) 3, universal access to contraception should be ensured by 2030.^2,11,14^ However, previous studies show that adolescents have an unmet need for contraception despite wanting to be better informed about sex and the implications thereof.^5,15,16^ This has been attributed to various factors that negatively influence or impede access to contraception among adolescents. These factors include judgemental attitudes from some healthcare providers towards young people, adolescent misconceptions about the use and effects of contraceptive methods, cultural or religious opposition, providers’ bias against some methods, and limited knowledge about contraception.^15,16,17,18,19^ This suggests that there are challenges with contraception access for this age group.

A systematic review comprising 15 studies looked at predictors of pregnancy among young people in sub-Saharan Africa.^20^ As found in various studies, the most cited predictors of adolescent pregnancy included sexual coercion from male partners, low or incorrect use of contraceptives, a lack of parental communication and support, or poor parenting.^5,20,21,22^ Another systematic review comprising 20 studies focused on accessing and utilising adolescent and youth-friendly SRH services.^23^ The findings of this study revealed that the most common barriers to access were the negative attitude of healthcare providers, inconvenient hours, quality of services, unskilled healthcare providers, and limited knowledge and awareness about the services among adolescents or youth as found in other studies.^4,18,23^ In addition, the study found that community outreaches, school health education, peer-led education and mass media campaigns, and sports and entertainment activities at youth centres facilitate access to SRH services.^23^

Much of the existing research on SRH-related issues has been predominantly conducted by examining the perspectives of healthcare providers, adolescent girls and young women or sexually active women of reproductive age in urban areas.^17,24,25^ Studies on adolescent access to contraception that have been conducted in both urban and rural primary healthcare (PHC) settings are scant. Building on existing research,^17,18,23,26^ this study explores factors that influence adolescents’ access to contraceptive methods from the perspective of healthcare providers working in rural and urban public PHC clinics.

Based on the aim of this study, this study adapted the ecological model as a theoretical framework. According to the ecological model, multiple factors affect an individual’s behaviour.^27,28^ The model highlights how adolescents are nested within different spheres or levels of influence that comprise family, peers, community, culture and government systems and policy. The first sphere represents the individual and includes behavioural characteristics. This is followed by relationships (e.g. with providers) and reflects the interaction between adolescents and family members, intimate partners, and peers.^27^ The outermost sphere characterises the interaction between the individual and community (e.g. schools and clinics), and lastly, society (e.g. healthcare policy and media).^27^ The diversity of influences among spheres ultimately shapes adolescents’ behaviour in South Africa. Using this model will give a better understanding of what influences access to contraceptive methods among adolescents. Decision-makers from health departments could use the findings of this study to inform strategies to facilitate access to contraceptive methods among adolescents in South Africa. In time, this could help address challenges experienced by healthcare providers and improve access to contraceptive methods among adolescents in South Africa.

## Research methods and design

### Study design

This study utilised a descriptive exploratory qualitative research design because it is valuable in exploring the real-life perspectives of participants.^29^ The study followed the Consolidated Criteria for Reporting Qualitative Studies (COREQ) guidelines for reporting the findings.^30^

### Setting

**FIGURE 1:**
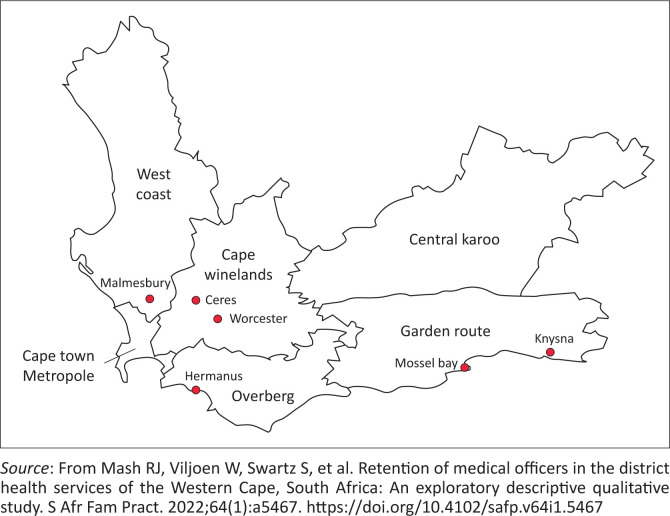
Western Cape health districts.

The South African health system consists of the National Department of Health, nine provincial health departments and 52 health districts. This study was conducted in two health districts of the Western Cape province in South Africa. That is the City of Cape Town Metropolitan Municipality (Cape Town Metropole) and West Coast district. It is estimated that there are 7.1 million people in the Western Cape province, and approximately 75% of them are served by public health services.^31^ About 66% of the people in the province reside in the urban *Cape Town Metropole*.^32^ The rest of the people are distributed in the other five rural districts in the province, as shown in Figure 1. Khayelitsha-Eastern, Northern-Tygerberg, Western-Southern, and Klipfontein-Mitchells Plain are the health subdistricts in the *Cape Town Metropole*.^31,32^ The West Coast district comprises five subdistricts: Swartland, Bergrivier, Matzikama, Cederberg and Saldanha Bay.^31,32,33,34^ Each subdistrict has healthcare clinics managed by *Provincial Health* (the provincial government) or *City Health* (the municipality or local government).

### Study population and sampling

The target population was providers working in PHC clinics in the Western Cape province. Primary healthcare clinics are nurse-based, doctor-supported clinics and community health centres available within a 5-kilometre radius to more than 90% of the population.^35^ At the time of the study, the Western Cape province had two health authorities managed by either the provincial or local governments. The provincial government had 54 fixed clinics, 34 community day centres and 11 community health centres.^31^ Under the authority of the local government, there were 69 clinics and 14 community day centres.^31^

The study used purposive sampling to recruit participants who had the potential to provide in-depth information because of their experience and availability.^36^ Following Tolley et al., the authors planned to recruit at least 10 providers in the urban district and 10 providers in the rural district (p. 60).^36^ Participants who met the following selection criteria were recruited: (1) a professional or enrolled nurse, (2) employed by the Provincial Health or City Health, (3) working in a PHC clinic, and (4) offering contraception services.

Permission to conduct the study was sought from Provincial Health and City Health, who confirmed the list of eligible PHC clinics. Next, the authors contacted subdistrict and clinic managers to inform them about the study and request permission to conduct it. Communication was performed through email and telephone. With the help of the clinic managers, one or two nurses from each selected PHC clinic were invited to participate in the study.

### Data collection

Data collection commenced from December 2019 to January 2020 but it was halted because of the COVID-19 and delays in getting authorisations from the province. In April 2022, data collection was restarted and concluded in June 2022. All the authors have experience in qualitative research. The authors comprise a female doctoral candidate, two female faculty members, and one male faculty member. The first author (T.K.), a doctoral candidate, conducted the interviews. O.A. and G.B.B. provided supervision.

The authors used an open-ended semi-structured interview guide, which was developed based on relevant literature.^27,28,36^ The interview guide incorporates four nested levels of the ecological model, as outlined in Baroudi’s conceptual framework on healthcare access.^28,37^ This guide explores policy, health system, community, and personal-level influences through various questions about SRH services, knowledge about contraception, perspectives on adolescents’ access to contraception, and the challenges they face.

Interviews were held in a private room at the healthcare clinics where participants worked at a time chosen by each participant. The interviews did not disrupt patient care or the operations of the healthcare clinics. All interviews were conducted in English because participants could speak and understand the language. To enhance trustworthiness and rigour, the authors established rapport with the participants before the interviews.^38^ During data collection, participants were asked for clarification when needed. In addition, the authors paraphrased and summarised points that participants discussed during and at the end of each interview. T.K. took handwritten field notes, which included a record of decisions taken during data collection. The participants agreed to be audio recorded. The interviews took 30 to 60 minutes. Data collection was concluded when the authors no longer received new information. The authors had no relationship with the participants before collecting data for this study. Therefore, there was no conflict of interest with the participants.

### Data analysis

The interviews were transcribed verbatim by an experienced research assistant and checked by the first author (T.K.). Using the process of thematic analysis described by Braun and Clarke, the authors familiarised themselves with the data by listening carefully to the audio-recorded interviews, reading the transcripts, and comparing them to their field notes.^39^ The transcribed interviews were imported into ATLAS.ti 22 (qualitative analytic software). After transcribing the interviews verbatim, the authors read the typed transcripts several times, checked them for accuracy, generated the initial codes and searched for themes and subthemes. T.K. and O.A. discussed the codes, themes, and sub-themes to avoid bias and strengthen the credibility of the analysis.^40^ Next, the themes were reviewed, defined, and named before producing the final report. In addition, the research process was reported as precisely as possible.^30,40^ The authors (T.K., S.M., G.B.B. and O.A.) engaged in discussions and reflected on personal experiences, views, and cultural backgrounds, which facilitated reflexivity in this study. T.K., G.B.B., and O.A. discussed the nature and implementation of the study and ratified it.

### Ethical considerations

An application for full ethical approval was made to the Biomedical Research Ethics Committee of the University of the Western Cape and the Western Cape Department of Health and ethics consent was received in 2019 and extended to 2021 and 2022. The ethics approval number from the University of the Western Cape is BM19/1/24, and the ethics approval number from the Western Cape Department of Health is WC_201905_040. All the participants provided written informed consent after receiving an explanation of the study’s nature, purpose and procedure. The data were encrypted and stored in password-protected computers to ensure anonymity and confidentiality.

## Results

### Participants’ characteristics

Twenty-four nurses agreed to take part in the study. All the participants were biologically female and reflected the racial groups found in South Africa. The authors interviewed clinic managers and professional and enrolled nurses who provide contraception (or family planning) services. Clinic managers are qualified professional nurses who manage their respective healthcare clinics.^9^

Table 1 shows the socio-demographic characteristics of the participants.

**TABLE 1:** Socio-demographic characteristics of the participants.

Participant characteristics	*n*	%
**Gender**		
Male	0	0
Female	24	100
**Racial group**		
Black African	10	42
Mixed race	6	25
Indian	1	4
White	7	29
**Designation or current position**		
Clinic managers	5	21
Professional nurses	13	54
Enrolled nurses	6	25
**Health authority**		
Provincial health	18	75
City health	6	25
**District (locality)**		
City of Cape Town (urban)	13	54
West Coast (rural)	11	46
**Total**	**24**	**100**

Based on the analysis, four overarching themes captured participants’ perspectives about adolescents’ access to contraceptive methods. These are personal, community-level, health system and policy-level influences. These themes are unpacked in the following sub-sections. Table 2 shows an overall picture of the themes produced during data analysis.

**TABLE 2:** Depiction of the thematic areas subtracted from the analysis.

Theme	Sub-theme
Personal influences	Adolescents’ level of awareness
	Attitudes
Community-level influences	Home environment
	Sociocultural norms
	Social circle
Health system influences	Availability of preferred methods
	Bias in method promotion
Policy-level influences	School policies
	Human resources for health capacity

### Personal influences

According to the participants, the ‘level of awareness’ and ‘attitudes’ (of adolescents and providers) influence access to contraception among adolescents.

#### Adolescents’ level of awareness

Participants indicated that adolescents have limited knowledge about contraception, especially at first contact. They stated that it is essential for adolescents to learn about available methods, the benefits of using contraceptive methods, and how to access contraception services:

‘[*T*]hey don’t have much information about contraceptives and the condom use … (Some) are not using the condom and do not understand the importance of using a condom because now our numbers are increasing for those who are testing HIV positive …’ (Interview 020, professional nurse, urban area)

Of primary concern was boys’ level of awareness about contraception. Participants felt that it was lacking compared to girls. To emphasise the point, participants shared that some boys do not know that they can prevent unintended pregnancies. This is because boys do not regard condoms as a contraceptive method but as a protective barrier from STIs:

‘… boys will say they use condoms to prevent STIs and HIV. They don’t think of pregnancy … They think there is nothing they can do to prevent pregnancies.’ (Interview 023, professional nurse, urban area).

Participants highlighted this as an area for improvement because it negatively influences boys’ ability to access contraception services. As such, participants recommended that there should be educational strategies about contraception for boys (and men):

‘I think we need to focus more on boys only. Because when we speak to them, they do not know. If they come here and we ask them if the girlfriend is on family planning. Their answer is, I don’t know or I’m not sure. If you ask them when last the girlfriend got the period. They don’t know or they are not sure. So I think they need to be educated also about family planning. Maybe that way, we can reduce teenage pregnancies. Because there will also be knowledge on the other side.’ (Interview 023, professional nurse, urban area)

Participants also acknowledged that myths and misconceptions about specific contraceptive methods influence adolescents’ access to contraceptives. One of the misconceptions is that a hormonal contraceptive method prevents the risk of pregnancy and STIs – as if it has a dual role:

‘For them they think that when they use contraceptives, they are doing everything, which is not the case.’ (Interview 020, professional nurse, urban area)

Other myths and misconceptions came from not understanding how contraceptives work in the body, and what their benefits are. Participants felt providers could dispel such myths and misconceptions by clarifying and sharing accurate information about the side effects:

‘We tried to promote the implanon but you just mention the implanon and they say they don’t want it. I ask why and you will hear someone saying the hair is falling out and the other one say the thing is moving around. So, it is all hearsay [*gossip*], and they don’t come and find out what is the real thing and any contraception has side effects but it’s not going to say is going to happen to you but to explain everything to them.’ (Interview 01, enrolled nurse, rural area)

Another aspect related to awareness that was of concern to participants is comprehension. They were of the view that adolescents’ ability to grasp information would help them with access (and utilisation) of contraception. However, some adolescents struggled to understand or synthesise information:

‘… [*A*]nd some of them you can even see when you are talking to them that no she does not understand what you are talking about … They are not able to comprehend the information. They can hear the information you’re chatting with them, but they can’t really process it or understand it fully.’ (Interview 021, enrolled nurse, urban area)

One participant gave an example by highlighting a distinction between accessing SRH information and utilising it (or changing behaviour):

‘The information, I’m not even sure if it sinks in. because the same person that was here saying they have three partners, when they come back a month after with the same thing, they will have four and not three anymore …’ (Interview 003, professional nurse, urban area)

Some participants were worried about not having information booklets, pamphlets, and other educational material in clinics, local churches, schools, television, and social media. They stressed that continuous messaging and simplified reading material can improve adolescents’ access to and understanding of information. This would then contribute to improving adolescents’ level of awareness about contraception:

‘We do outreach at schools … Yet, we don’t even have pamphlets to give out and they just don’t get it. Maybe even that, um, yes.’ (Interview 011, professional nurse, rural area)

Despite all of that, participants were confident that there is value in ensuring that adolescents have access to contraceptives. They felt that adolescents have an appreciation for contraception services when they are familiar with them. They indicated that the uptake of contraceptive methods by adolescent girls is because:

- They are sexually active and want to reduce the risk of unplanned pregnancies. This also applies to adolescent girls who started using contraceptives after pregnancy:
  ‘The value is that, um, because just now they are sexually active, and there are societal influences … they can access it and prevent unwanted pregnancy.’ (Interview 018, professional nurse, urban area)
- They receive helpful information from an objective source, which helps them make informed decisions:
  ‘I think the teenagers and school-going girls that do come and arrive at facilities … they … they might … they give themselves an opportunity and a fighting chance to get to speak to a professional, knowledgeable person … It gives, it opens the door for that youngster to go and have accurate information, support, knowledge about their bodies, so on.’ (Interview 002, clinic manager, urban area)
- They can potentially stop menses (getting periods) if they opt to:
  ‘So you might have a situation where, and I’ve had that before, where … a young girl, 14 or 13, just having menarche ends up with a sister saying I’m bleeding and I don’t want this, and I heard that if I go on the pill I won’t have this, or I heard this and this.’ (Interview 002, clinic manager, urban area)
- They can talk openly with providers, at the clinic or elsewhere:
  ‘We really go to the schools, and we go to the churches. We talk to the teenagers, … we are trying to provide them with all the information, and I don’t know why there is so much teenage pregnancies.’ (Interview 015, clinic manager, rural area)

#### Attitudes

Participants reported that adolescents display a negative attitude in various ways. Some participants stated that adolescents lack interest when a provider seems prescriptive or avoids having a serious conversation during consultations. This happens even if an adolescent misses a prior ‘family planning’ appointment:

‘They don’t like to be told by a nurse what to do because they want to feel in control, they are informed and think like how this person can tell me this.’ (Interview 013, enrolled nurse, rural area)

‘It is not easy because sometimes they do not come on their date. They would come late … and it is not easy. If you are talking to a teenager, they are always playing thinking that you are playing, or you are joking.’ (Interview 021, enrolled nurse, urban area)

Negative attitudes are also perceived as ignoring or distrusting advice offered by healthcare providers:

‘That thing of nurses shouting to clients, no it is no longer happening. So now they don’t trust what you are telling them … but we are telling them that we are the ones who are starting them on contraceptives. You are the one who came to us asking about what we are providing so if you’ve got a problem or question you must come and ask us so that you can explain to you and give you more information, not for you asking your mother.’ (Interview 020, professional nurse, urban area)

‘The clients have attitudes towards nurses in many times and we can’t do anything about this. For example, a client just refused to use anything. I’ve given her all the information and after you had a nice talk but still refuse and you will get the nurse attitude. You find that this girl is so stubborn, and you want to help her, and she doesn’t want to be helped.’ (Interview 015, clinic manager, rural area)

On the other hand, a few participants acknowledged that some healthcare providers have a negative attitude towards clients:

‘We cannot just point the fingers here just to clients because we also have our own attitude for as much as the nurses got attitude also, we do get attitude from the clients … client defaulted in 2019 when you ask the client about why and then the client would say because I did not have time to come to the clinic. So you see those kind of attitude and then after that, you won’t say anything. You’ll just give a client whatever he came for and then they are off and go because I did not have time.’ (Interview 019, professional nurse, urban area)

Some of the participants attributed providers’ negative attitude to feeling frustrated because of how some adolescents communicate or ignore health advice provided to them:

‘The nurses too, do have an attitude. But the attitude of the nurse is mainly because of the frustrations.’ (Interview 014, clinic manager, rural area)

Most participants reported that healthcare providers’ feelings of hopelessness and frustration do not affect the services they offer to adolescents. In addition, the participants indicated that it is essential for healthcare providers to be open-minded because they do not know everything about their adolescent clients:

‘They come here … and take implanon. The next day they don’t want implanon, and you ask what is it that you want. Because obviously they don’t want condoms. So, there are a lot of social issues we are not aware of that the youth have to go through …’ (Interview 0120, clinic manager, urban area)

### Community-level influences

In this study, the authors found that the home environment, sociocultural norms, and the social circle of the adolescent influence access to contraception.

#### Home environment

The participants highlighted how the home environment influences adolescents’ access to contraception. The authors found that participants consider the home environment of their adolescent clients. For example, whether there is open communication, support, and guidance at home. They also consider the physical or structural environment (which could be characterised by a high unemployment rate in the community, poor living conditions or dysfunction). This is what one participant shared:

‘Most of the parents is on drugs, so there’s that form of neglect and then they need to rely on others … There’s no encouragement, no engagement [*at home or in the community*]).’ (Interview 003, professional nurse, urban area)

Thus, they also consider if their adolescent client is exposed to alcohol or other substances and their living arrangements. This enables the provider to focus on the client during consultation, build rapport, and not be insensitive or oblivious. This also allows adolescents to access the services they need.

Participants cited examples of girls who were brought by either their mothers or older sisters for contraceptive methods without any guidance or discussion with the adolescent child. It is as if the family or parents’ perceptions of right and wrong dictate what the adolescent does:

‘I have had many of those where I have to sit the mother who is 30 years old, and the child is 12. So, there’s issues. But the issues have little to do with the child but more with the parent who doesn’t want her child to get pregnant like she did when she was 17 or 18 …’ (Interview 002, professional nurse, urban area)

‘Like one was brought by her sister because the sister was saying she is naughty. The girl didn’t even know. So, the sister brought her in because she gets home late. Sometimes the community is more judgmental than positive. So, I think the family is influential.’ (Interview 007, enrolled nurse, urban area)

#### Sociocultural norms

The participants reported that sociocultural norms also affect adolescents’ access to contraception services. In communities where it is frowned upon to provide contraception to unmarried young people, it becomes challenging for adolescents to visit a clinic for contraception:

‘It is complicated because there are also cultural beliefs, there are religious beliefs, there are … but at the end of the day the cultural and religious beliefs are yours as a parent, not necessarily those of the child.’ (Interview 0120, clinic manager, urban area)

This also applies to adolescents who come from conservative religious (family) backgrounds where girls may be judged harshly for using contraceptives or being sexually active:

‘[*Y*]ou know about our culture when people are using family planning, and they label you as a loose girl other than teaching the kids about how to deal with your body. And I told the other lady the other day that there is no way that you can get rid of this family planning because it is nature as you are growing now, and your body is starting now wanting to have sex. It is not something that you can just cut off.’ (Interview 0060, professional nurse, rural area)

Some of the participants shared that in some communities it is common for boys and men to have multiple partners and not use condoms. This exacerbates the risks of unplanned pregnancies and STIs:

‘The promiscuity, cultural issues … I don’t know … so … men is allowed to have more women. That to me is a very big problem … So I always tell them – you are having 4, so how many partners does each of them also have? Because if you are not in a relationship, you are just having intercourse, the other person can also think that I can also have a relationship. So it’s not just you having four people, the people might be having partners as well. There is a lot of cultural view that males can have more females because it is allowed in the culture.’ (Interview 003, professional nurse, urban area)

#### Social circle

The authors learned from the participants that adolescents are greatly influenced by peer pressure. Participants reported that girls tend to seek the contraception methods used by their friends (even before learning from a healthcare provider about the different options that could be made available to them):

‘Most of them, I think it’s … their friends. Peer pressure plays a role.’ (Interview 023, professional nurse, urban area)

This was apparent with other behaviour, such as the early onset of sexual behaviour:

‘I would have intimate talks with the youth, and I would how did you get to the decision of having intercourse at 13 or 14 years old. It’s always a friend. It’s always a friend. So I think our youth has become so dependent on a friend or what a friend says. Yet we don’t always have good friends.’ (Interview 003, professional nurse, urban area)

Participants also shared that girls like to go to their clinic appointments as a group. Thus, there is a strong sense among adolescents to do things collectively. It might be easier for adolescents to conform and be admired than risk being singled out and ridiculed by their peers:

‘[*A*]nd they will come like they are three or four together.’ (Interview 013, enrolled nurse, rural area)

Some of the participants reported that sexual partners (or boyfriends) also influence girls’ access to contraception. The participants felt this was more common when the boyfriend was much older (an adult) than the girl (an adolescent):

‘They come because the boyfriend said so … And there is one waiting outside.’ (Interview 0060, professional nurse, rural area)

This could be attributed to the age gap and the power dynamics caused by that. Thus, the older boyfriend of an adolescent girl will decide whether she uses a contraceptive method or not, which method she uses, and if she should get pregnant:

‘I had an encounter. It was a teenager. She was 17 [*years old*]. So, she came for family planning but later on I found out she wanted a pregnancy test. Because pregnancy testing is part of the [*initial*] family planning consultation. So, I tested her and found that she was not pregnant, and then I told her which family planning methods are available. Then she said no she is not interested in family planning at the time. So I asked her if she is using a condom. And she said no. so I warned her that she could get pregnant if she is not using family planning … She was an HIV positive patient. So, I asked her if she does not want to take family planning … so that she gets accustomed to ART and then consider it later if she changes her mind … she said no, her partner wants a baby … That thing of trying to assist and advise a child, but then she insists that she wants to have a child.’ (Interview 001, professional nurse, urban area)

### Health system influences

The participants shared what they consider potential health system-level challenges from their perspective and that of their respective clients. These include the availability of preferred methods and bias in method promotion.

#### Availability of preferred methods

Participants confirmed that contraceptive methods are available in clinics at no cost. These include both the progesterone-only Depo-Provera injectable, also known as ‘the 3-month injection’, and Nur-Isterate (or ‘the 2-month injection’), male and female condoms, the implant (Implanon), and emergency contraception. Thus, in terms of access, the affordability of contraception is not a concern for adolescents.

However, during the COVID-19 pandemic, a few participants encountered a shortage of preferred contraceptive methods. Because of this, access to contraception was affected temporarily:

‘It was only when COVID started, we had an issue. But it was a once-off situation. It does not happen that we are out of stock. Even so, if we have a stockout issue, we are able to borrow from other clinics [*as a temporary measure*]. So availability is not an issue.’ (Interview 023, professional nurse, urban area)

Participants indicated that when a preferred contraceptive method is unavailable, adolescents must go to another clinic or return on another date. Unfortunately, this creates a gap in access and may place adolescents at risk:

‘Everything that they would want they’re supposed to get it here. But some of the things are beyond us and we can’t do anything about that. Because even for us, it is not nice because they’re coming back from school, you see, they’ve been in school for the whole day, and they must still walk to the facility, and when they come here, we can’t provide them with the service that they want, and then they must walk to another facility across the bridge, and they must walk to that side so it is a challenge. As a result, some of them who just say ok let me have what you have here and they would go back to the injectables or oral contraceptives and later they can go for what they’ve been wanting.’ (Interview 020, professional nurse, urban area)

#### Bias in method promotion

Although oral contraceptive pills and intrauterine devices (IUD) were available methods at the visited clinics, participants reported that they are offered to older clients. The providers encourage adolescents to use the injectables or implants because it is a long-acting reversible contraception (LARC):

‘All sorts of methods are easily available. It’s just that IUD they don’t like it – they don’t like it much. The loop, that is. And of course, they are young, and maybe we don’t advocate for that when it comes to the youth. The implant is the one we are really advocating for, because they forget and do not honour their appointments for oral. Whereas implant is for three years and after three years you can take it out. We try to minimise teenage pregnancy. So that is the is the most favourable one, which they do not like, I must say.’ (Interview 0120, clinic manager, urban area)

‘We are not very fond of using tablets for the young girls especially on schools. Once they are at the college or start working then we start treating them with a better option. But there are few teenagers that feels like she wants the pill then we give her the pill but you must understand that she will take a responsibility for that … But mostly we promote the injection just for them to remember for the client’s part.’ (Interview 008, clinic manager, urban area)

### Policy-level influences

The participants reported that school policies and human resources for health (HRH) influence adolescents’ access to contraception.

#### School policies

Most of the participants reported that school policies influence adolescents’ access to contraception. It was found that certain high schools allow adolescent girls to access contraceptives during school hours. In such cases, there is a formal agreement between the school and the local clinic:

‘Every Friday I am going to [*name of the high school*], so I’m dealing with those from 2000, um, from the 2006 upward … and there are no male clients …’ (Interview 021, enrolled nurse, urban area)

However, most of the participants reported that they are not allowed to offer contraceptive methods to learners (school-going adolescents) at school premises or during school hours. School policies prescribe this:

‘So a lot of people is opposing it [*contraception*]. Even teachers are opposing it [*contraception*]. So it gives you that picture that … it gives students that negative “what is the teacher going to think of me” picture. Because if my teacher thinks it’s wrong, I shouldn’t be using it, but I am having sex anyway …’ (Interview 008, professional nurse, urban area)

#### Human resources for health capacity

Clinics should have an adequate number of well-trained healthcare providers. This enables adolescents access to contraception. A few of the participants confirmed this. They mentioned that when healthcare providers cannot administer all the preferred contraceptive methods, there is an interruption in access to care:

‘The patient can be told, sorry I can’t assist you today, you have to come back when the trained sister is in, you have to make a booking. Or I need to refer you to another facility where I know there is somebody trained. So it does influence …’ (Interview 002, clinic manager, urban area)

Another participant also shared that understaffed clinics make it difficult for clients to receive comprehensive care, as there may be inadequate consultation time:

‘There are policies in place, but our main problem is human resources … Sometimes, it’s like the government is putting money in the wrong place. A few years back we had our municipality clinics and we provided services and at times we wouldn’t have electricity, but we managed. We had more nurses but nowadays they scale down on the staff …’ (Interview 008, clinic manager, rural area)

## Discussion

This study explored the perspectives of healthcare providers about the factors that influence access to contraceptive methods among adolescents in South Africa. The findings reveal that various factors influence access to contraception among adolescents. Personal, community, health system, and policy-level influences can facilitate or impede access to contraceptive methods among adolescents.

According to healthcare providers, adolescents’ level of awareness influences their ability to access SRH services and, ultimately, contraceptive methods. In this study, healthcare providers revealed that knowledge and awareness about contraceptive methods improved among adolescents after consultation (with healthcare providers). The authors also learned from healthcare providers that through exposure to correct information, adolescents gain access to contraception and other healthcare services. In addition, healthcare providers found that the level of awareness about contraception differs between adolescent boys and girls. Research shows that adolescent girls tend to be more knowledgeable about contraception compared to adolescent boys.^21^ Adolescent boys’ level of awareness about contraception is concerning. But this could be attributed to health policies that focus mainly on girls and women and educational messaging on access to contraception.^11,41^ Shabani pointed out that male adolescents are exposed to information reinforcing poor gender norms. This contradicts the SDG targets of ensuring universal access to SRH services, including quality essential family planning services and access to safe, effective, quality and affordable essential medicine.^1,2,11^ The Ministry and Departments of Health should include adolescent boys in SRH policies and interventions. This approach could improve access to contraception services, ensuring that both adolescent boys and girls have access to information about contraceptive methods.

Adolescent girls’ access to contraception is influenced by the networks closest to them. A study conducted in Kenya by Zinke-Allmang and colleagues found that one’s mother, sister, or boyfriend were key influencers of adolescent SRH choices.^22^ Trust and the ability to maintain the secrecy of contraception use were of utmost importance to adolescents to reduce their susceptibility to stigma.^22^ In this study, healthcare providers reported that mothers and peers influence adolescent girls’ first visit to a clinic and access to contraceptives. However, the root of it might be fear. It may be fear of parents’ rules and expectations, community judgement, stereotypes and stigma, unintended consequences such as adolescent pregnancy and contracting an STI, losing a sexual partner, or potential side effects.^21,22,23,42^ It could be beneficial for the health districts and PHC clinics to conduct community outreach campaigns to raise awareness about the importance of access to contraception for adolescents and the risks of not using contraception. This could be done in partnership with the Department of Social Development, churches and religious groups to reach parents and community members. Prominent or influential people could also implement these campaigns on various media platforms to reach adolescents and other stakeholders.

This study suggests that sociocultural norms influence adolescents’ access to contraception. The authors learned that it is socially acceptable for adolescent boys to have multiple sexual relationships. This is supported by research conducted in other countries.^21,43^ On the other hand, some adolescent girls engage in intergenerational relationships, believing that this brings economic benefit and relief to their circumstances.^37^ In both scenarios, risky sexual behaviour comes into play, leading to unintended pregnancies among girls and STIs among boys. In both scenarios, adolescents are predisposed to harmful social risks and poor health outcomes.^23,43^ It also reflects a gender power dynamic, with boys and older men overpowering adolescent girls’ ability to make decisions.^20,42^ A recent analysis of data from South Africa reported high rates of pregnancies among girls between the ages of 10 and 14.^5^ This raises questions about how adolescents understand sexual coercion and the scope of SRH programmes. It also reveals the need for government departments (such as the Departments of Health, Basic Education, Social Development and the National Youth Development Agency) to implement contraception-related interventions that target different age groups and sociocultural norms. This could help minimise harmful gender norms.

Previous studies have highlighted the challenges faced by adolescents who need to access contraception,^5,26,44^ as discussed in this article. This article also points out the limitations of PHC clinics for school-going adolescents. From a policy perspective, the findings of this study suggest that school-going adolescents experience constraints in their pursuit of access to contraception. This requires an exploration of school policies and teachers’ perspectives. To explore the perceptions of teachers and parents, one needs to take into cognisance the message this gives adolescents. For example, are teachers and parents saying contraceptives are harmful? If so, this could lead to shame and guilt among adolescents. But it also contradicts messaging that advocates for universal access to contraception and prevention of adolescent pregnancies. It would be useful for future research to investigate the efficacy of contraception-related interventions. In addition, future research could examine strategies to strengthen the provision, access, and utilisation of contraception among young men and women.

The authors recommend that the public sector introduce a multisectoral (government, civil society, the private sector, and development partners) educational campaign targeted at adolescents and parents. The campaign should use short, direct, and visual messaging through social media platforms.^23^ The Department of Health and the Department of Basic Education should work closely with youth agencies and find innovative ways of strengthening access to contraception among young people. This could include making contraception available at youth agency offices and provincial and municipal buildings.

## Limitations

This study has a few limitations. Firstly, this study was conducted with providers working in PHC clinics in one urban area and one rural district in the Western Cape province. Therefore, the transferability of the findings is limited to similar contexts and settings.^36^ Secondly, the data collection did not include users or adolescents, thus reflecting only the views of providers. Moreover, participants could have provided socially desirable responses or tried to conform to perceived expectations when responding to interview questions. Lastly, while the participants were proficient in English, it is important to note that English is not their first language. For some of them, their first languages are Afrikaans and isiXhosa. This could have affected how they responded to the questions.

## Conclusion

Adolescents’ access to contraception is a gateway to reducing unplanned pregnancies and enhancing adolescents’ SRH. Parents, peers, sexual partners, and healthcare providers all have some level of influence on adolescents’ ability to access contraception methods. These include level of awareness about contraception services, home environment, and health system challenges such as provider biases and school policies.

To contribute towards universal access to contraception, factors that negatively influence adolescent access should be addressed. Health departments and organisations should make efforts to strengthen adolescents’ access to contraception. Strategies that are relatable to adolescents should thus be developed to enhance access. Educational campaigns using different media platforms could be used to improve awareness and access to contraceptive methods.
